# Supplementary material for: Changes in diet, exercise and psychology of the quarantined population during the COVID-19 outbreak in Shanghai
Source: PLoS One. 2023 Aug 2;18(8):e0284799. doi: 10.1371/journal.pone.0284799 (PMC10395905; doi:10.1371/journal.pone.0284799)
Supplement: Attachment — Submitted filename: Review_ PONE-D-22-2437.docx [file pone.0284799.s001.docx]

This study reports on changes in the diet, physical activity levels, and psychological status of the quarantined population during the COVID-19 outbreak in Shanghai, China. While the premise of this study is relevant and looks important, there are some comments/suggestions that the authors need to address before resubmission.

1. The background statement in the abstract should summarize the latest information on the topic; key phrases that pique interest, which lead to a clear definition of the study objectives. This is currently lacking.
2. I see that the authors have compromised on the results section of the abstract, which may be due to the word limits. As you have several significant findings, please, make sure you included as many as you possibly can, and consider mentioning statistical indicators.
3. There is little baseline knowledge on dietary, physical activity, and psychological status changes in the introduction. Authors need to develop further context and provide more information in the context of the previous studies and explain why their research is important. You may find the following studies helpful.

- Jia P, Liu L, Xie X, Yuan C, Chen H, Guo B, Zhou J, Yang S. Changes in dietary patterns among youths in China during COVID-19 epidemic: The COVID-19 impact on lifestyle change survey (COINLICS). Appetite. 2021 Mar 1;158:105015. doi: 10.1016/j.appet.2020.105015.
- Elnaggar RK, Alqahtani BA, Mahmoud WS, Elfakharany MS. Prospective analysis of physical activity levels and associated fitness factors amid COVID-19 pandemic and social-distancing rules. A special focus on adolescents. Sci Sports. 2022 Apr;37(2):131-138. doi: 10.1016/j.scispo.2021.07.002.
- Yu B, Zhang D, Yu W, Luo M, Yang S, Jia P. Impacts of lockdown on dietary patterns among youths in China: the COVID-19 Impact on Lifestyle Change Survey. Public Health Nutr. 2021 Aug;24(11):3221-3232. doi: 10.1017/S1368980020005170.
- Elnaggar RK, Alqahtani BA, Mahmoud WS, Elfakharany MS. Physical Activity in Adolescents During the Social Distancing Policies of the COVID-19 Pandemic. Asia Pac J Public Health. 2020 Nov;32(8):491-494. doi: 10.1177/1010539520963564.
- Yang GY, Lin XL, Fang AP, Zhu HL. Eating Habits and Lifestyles during the Initial Stage of the COVID-19 Lockdown in China: A Cross-Sectional Study. Nutrients. 2021 Mar 17;13(3):970. doi: 10.3390/nu13030970.
- Hossain MM, Tasnim S, Sultana A, Faizah F, Mazumder H, Zou L, McKyer ELJ, Ahmed HU, Ma P. Epidemiology of mental health problems in COVID-19: a review. F1000Res. 2020 Jun 23;9:636. doi: 10.12688/f1000research.24457.1.
- Dong L, Bouey J. Public Mental Health Crisis during COVID-19 Pandemic, China. Emerg Infect Dis. 2020 Jul;26(7):1616-1618. doi: 10.3201/eid2607.200407.

4. The 1^st^ paragraph in the introduction section is a bit long. Please revise for brevity, directness, and conciseness.
5. Once again, the introduction should end with a straightforward and clear statement of study objectives. This is crucial to help readers make proper inferences and follow other parts of the study based on the study objectives.
6. I would have liked it if the authors had merely offered a summary of the survey's questions in the Methods under the “Measures” subheading.
7. Although 625 respondents appear to be reasonable, I am not sure if this study is powered enough for the study design and outcome measures.
8. You looked at how the respondent changed their behaviors from before to after closed management. Does the independent t-test fit this analysis?
9. I am not clear how Figure 3 illustrates the difference in exercise mode before and after closed management.
10. Discussion - The authors should discuss in-depth, focusing on the interpretation of their results in the context of the previous evidence and the implication of the study findings.
11. How can health care authorities in Shanghai make use of the results presented herein to facilitate access to care and for policy-making? Consider commenting on this point in the “Discussion” section.
12. Motivate on the study's merits, limitations, and how cautious readers should be while interpreting the results and give suggestions for future work.
