# Supplementary material for: Changes in diet, exercise and psychology of the quarantined population during the COVID-19 outbreak in Shanghai
Source: PLoS One. 2023 Aug 2;18(8):e0284799. doi: 10.1371/journal.pone.0284799 (PMC10395905; doi:10.1371/journal.pone.0284799)
Supplement: Attachment — Submitted filename: Reply-Review_ PONE-D-22-2437.docx [file pone.0284799.s002.docx]

Dear Editor and reviewers,

Thank you for your letter which enclosed the reviewer’s comments concerning our manuscript entitled “Changes in diet, exercise and psychology of the quarantined population during the COVID-19 outbreak in Shanghai” (Research Article, PONE-D-22-24378). Those comments are all valuable and very helpful for revising and improving our paper, as well as the important guiding significance to our researches. We have studied comments carefully and have made correction which we hope meet with approval. The responds to the reviewer’s comments are as follows:

**Reviewer #3:** *This is my first time to review this paper. I have a few comments.*

**1. Comment:** *Under the Introduction, please mention how lockdown affect other countries besides China:*

*Search PubMed for: Partial Lockdown and Factors that were associated with an increased level of depression, stress, and anxiety were being single, separated, or widowed, a higher education level, a larger family size, loss of jobs and being in contact with potential COVID-19 patients*

**Response:**

We gratefully appreciate for your comment. We searched for the information you mentioned. In the introduction section, we add content about the impact of the lockdown on countries other than China.

The contents of the correction is as followed: A study in Vietnam found significantly higher levels of depression, anxiety and stress among people in lockdown due to being single, separated or widowed, higher levels of education, larger family size, unemployment, and contact with potential COVID-19 patients ^[19]^. A study from France says the lockdown affects people's mood, with hope and anxiety being the two ways to deal with uncertainty ^[20]^.

References:

19.Le HT, Lai AJX, Sun J et al. Anxiety and Depression Among People Under the Nationwide Partial Lockdown in Vietnam[J]. Frontiers in public health. 2020, 8: 589359.

20.Fouques D, Castro D, Mouret M et al. Perceptions of the Post First-Lockdown Era in the Current Covid-19 Pandemic: Quantitative and Qualitative Survey of the French Population. Frontiers in Psychology. 2021, 12. DOI: 10.3389/fpsyg.2021.668961

(The contents of the correction are highlighted in red in the Introduction on page 2 and References on page 15 of the manuscript.)

**2. Comment:**  *Under the discussion, please state limitation of this study as it did not measure COVID-19 burnout and discuss the following finding:*

*Search PubMed for: Burnout is an important public health issue at times of the COVID-19 pandemic. Current measures which focus on work-based burnout have limitations in length and/or relevance. When stepping into the post-pandemic as a new Norm Era, the burnout scale for the general population is urgently needed to fill the gap. This study aimed to develop a COVID-19 Burnout Views Scale (COVID-19 BVS) to measure burnout views of the general public in a Chinese context and examine its psychometric properties.*

**Response:**

We gratefully appreciate for your comment. In the "Discussion" part, we add some limitation of our study as it did not measure COVID-19 burnout. We searched and learned about the burnout you mentioned. These are also discussed in the "Discussion" section.

The contents of the correction is as followed: In the part of investigating the psychological state of the population under lockdown, this study did not involve the investigation of burnout in the population. Burnout is an important public health problem, and it is very important to study the burnout of the general public when entering the post-epidemic era.

References:

46.Lau SSS, Ho CCY, Pang RCK et al. Measurement of burnout during the prolonged pandemic in the Chinese zero-COVID context: COVID-19 burnout views scale[J]. Frontiers in Public Health. 2022, 10.

(The contents of the correction are highlighted in red in the Discussion on page 12 and References on page 17 of the manuscript.)

**3. Comment:** *The authors mentioned about psychological interventions and it must be online during the lockdown period. Please refer to the following:*

*The most evidence-based treatment is cognitive behaviour therapy (CBT), especially Internet CBT that can prevent the spread of infection during the pandemic.*

*Use of Cognitive Behavior Therapy (CBT) to treat psychiatric symptoms during COVID-19:*

*Mental Health Strategies to Combat the Psychological Impact of COVID-19 Beyond Paranoia and Panic. Ann Acad Med Singapore. 2020;49(3):155‐160.*

*Cost-effectiveness of iCBT:*

*Moodle: The cost effective solution for internet cognitive behavioral therapy (I-CBT) interventions. Technol Health Care. 2017;25(1):163-165. doi: 10.3233/THC-161261. PMID: 27689560.*

*Internet CBT can treat psychiatric symptoms such as insomnia:*

*Efficacy of digital cognitive behavioural therapy for insomnia: a meta-analysis of randomised controlled trials. Sleep Med. 2020 Aug 26;75:315-325. doi: 10.1016/j.sleep.2020.08.020. Epub ahead of print. PMID: 32950013.*

**Response:**

We gratefully appreciate for your comment. Thank you very much for providing these references. We have carefully read these articles and believe that their findings and results are of great help to our research and provide us with a lot of important information. In the introduction and discussion of this paper, we add the significance of the research findings of these articles to this paper and explain the importance of the research findings of these articles.

References:

18. Ho CSH, Chee CYI, Ho RCM. Mental Health Strategies to Combat the Psychological Impact of Coronavirus Disease 2019 (COVID-19) Beyond Paranoia and Panic[J]. Annals Academy of Medicine Singapore. 2020, 49(3): 155-160.

29. Zhang MWB, Ho RCM. Moodle: The cost effective solution for internet cognitive behavioral therapy (I-CBT) interventions[J]. Technology and Health Care. 2017, 25(1): 163-165.

45.Soh HL, Ho RC, Ho CS et al. Efficacy of digital cognitive behavioural therapy for insomnia: a meta-analysis of randomised controlled trials[J]. Sleep Medicine. 2020, 75: 315-325.

(The contents of the correction are highlighted in red in the Introduction on page 2-3, Discussion on page 11-12 and References on page 15-17 of the manuscript.)

Thank you again for your positive and constructive comments and suggestions on our manuscript. We hope you will find our revised manuscript acceptable for publication.

**NOTE:** All modifications to the manuscript are marked with red in the Word.
